# Electrical stimulation of somatic afferent nerves in the foot increases bladder capacity in neurogenic bladder patients after sigmoid cystoplasty

**DOI:** 10.1186/s12894-015-0023-8

**Published:** 2015-04-04

**Authors:** Guoqing Chen, Limin Liao, Di Miao

**Affiliations:** Department of Urology, China Rehabilitation Research Center, Beijing, 100068 China; Department of Urology, Capital Medical University, Beijing, China; Center of Neural Injury and Repair, Beijing Institute for Brain Disorders, Beijing, China

**Keywords:** Electrical stimulation, Foot, Bladder capacity, Neurogenic bladder, Detrusor overactivity

## Abstract

**Background:**

A previous study showed that foot stimulation can delay the bladder filling sensation and increase bladder volume in healthy humans without OAB. The aim of this study was to determine whether or not electrical stimulation of somatic afferent nerves in the foot can increase bladder capacity in neurogenic bladder patients after sigmoid cystoplasty.

**Methods:**

Eleven subjects underwent 30-min foot stimulation using skin surface electrodes connected to a bladder-pelvic stimulator. The electrodes were attached to the bottom of the foot. The subjects completed a 5-day voiding diary, during which time foot stimulation was applied on day 3. The stimulation parameter was a continuous, bi-polar square wave form with a pulse duration of 200 μs and a stimulation frequency of 5 Hz. The stimulation intensity was set by each subject at a maximal level without causing discomfort.

**Results:**

The volume per clean intermittent catheterization (CIC) was 279.4 ± 11.7 ml and 285.4 ± 11.8 ml on the 1st and 2nd days, respectively. On the 3rd day, the average volume per CIC increased to 361.1 ± 18.1 ml after stimulation (p <0.05). The average volume per CIC returned to 295.4 ± 13.4 ml and 275.1 ± 11.5 ml on the 4th and 5th days, respectively.

**Conclusions:**

Foot stimulation can delay the bladder filling sensation and significantly increase bladder capacity in neurogenic bladder patients after sigmoid cystoplasty.

## Background

Augmentation enterocystoplasty (AE) is the reference standard for patients with neurogenic bladder dysfunction (NBD) with the detrimental effects of high-bladder pressure on the upper urinary tract (UUT). The purpose of AE is to create a large-capacity, low-pressure, good-compliance reservoir with a preserved UUT, thus allowing for socially-acceptable continence [1]. Based on our previous research [1], we found that incontinence still occurred and bladder capacity was unsatisfactory within 6 months following sigmoid cystoplasty in some patients because of the weakened function of the sphincter, automatic contraction of the intestinal reservoir, and residual detrusor overactivity (DO) post-operatively. Therefore, we asked these patients to use oral anti-cholinergic agents until the bladder capacity increased and had good compliance.

A previous study showed that foot stimulation using skin surface electrodes inhibits DO and has a long-lasting effect in cats [2], likely as a result of stimulating branches of the tibial nerve in the foot. Recently, Chen [3] reported that foot stimulation can also delay the bladder filling sensation and increase bladder volume in healthy humans without OAB.

In the current study we reported the initial outcome of a clinical study in which we evaluated the effectiveness of electrical stimulation of somatic afferent nerves in the feet of neurogenic bladder patients who emptied the bladder by clean intermittent catheterization (CIC) after sigmoid cystoplasty.

## Methods

This study was approved by the Ethics Committee of the China Rehabilitation Research Center. All participants signed an informed consent. Foot stimulation was tested in 11 neurogenic bladder patients after sigmoid cystoplasty (7 males, 4 females; mean age, 28.9 ± 3.3 years; age range, 17–46 years) who used CIC to empty the bladder. All the patients were ≥ 1 month post-sigmoid cystoplasty (mean, 4.6 ± 1.2 months; duration, 1–12 months) for neurogenic bladder refractory to conservative treatment. Intra-operatively, a 20–30 cm segment of sigmoid colon was isolated with its vascular pedicle and opened on the anti-mesenteric border to form a patch. The detubulized sigmoid patch was sutured onto the opened bladder. After recovering from surgery, the capacity was increased from 105.6 ± 10.2 ml pre-operatively to 280.5 ± 11.7 ml by urodynamic evaluation. Then the patients were instructed to empty the bladder by CIC for life. Before surgery, all of the patients were diagnosed with incomplete spinal cord damage based on American Spinal Injury Association (ASIA) standards [4] and electrophysiologic assessment.

The subjects were instructed to record CIC volumes during a 5-day period without restriction of daily food and water intake when they returned to the hospital for a follow-up evaluation. The subjects were also instructed to perform CIC when urine leaked or in response to the usual bladder sensations in patients in whom bladder sensations still existed. Foot stimulation was applied for 30 minutes in the morning (9:00–9:30 a.m.) on day 3 with the subject in the sitting position. Two skin surface electrodes (4 × 4 cm) were attached to the plantar surfaces of both feet. A cathodal electrode was placed on the anterior aspect of the foot and an anodal electrode was placed between the inner foot arch and the heel. The two pairs of electrodes were connected to a bladder-pelvic stimulator [Bladder-Pelvic Stimulator (I) developed by Neural Electro-Mechanics Center of Chinese Academy Sciences and Dept. of Urology at China Rehabilitation Research Center, and supported by the China National Technology R&G Program].

**Figure 1:**
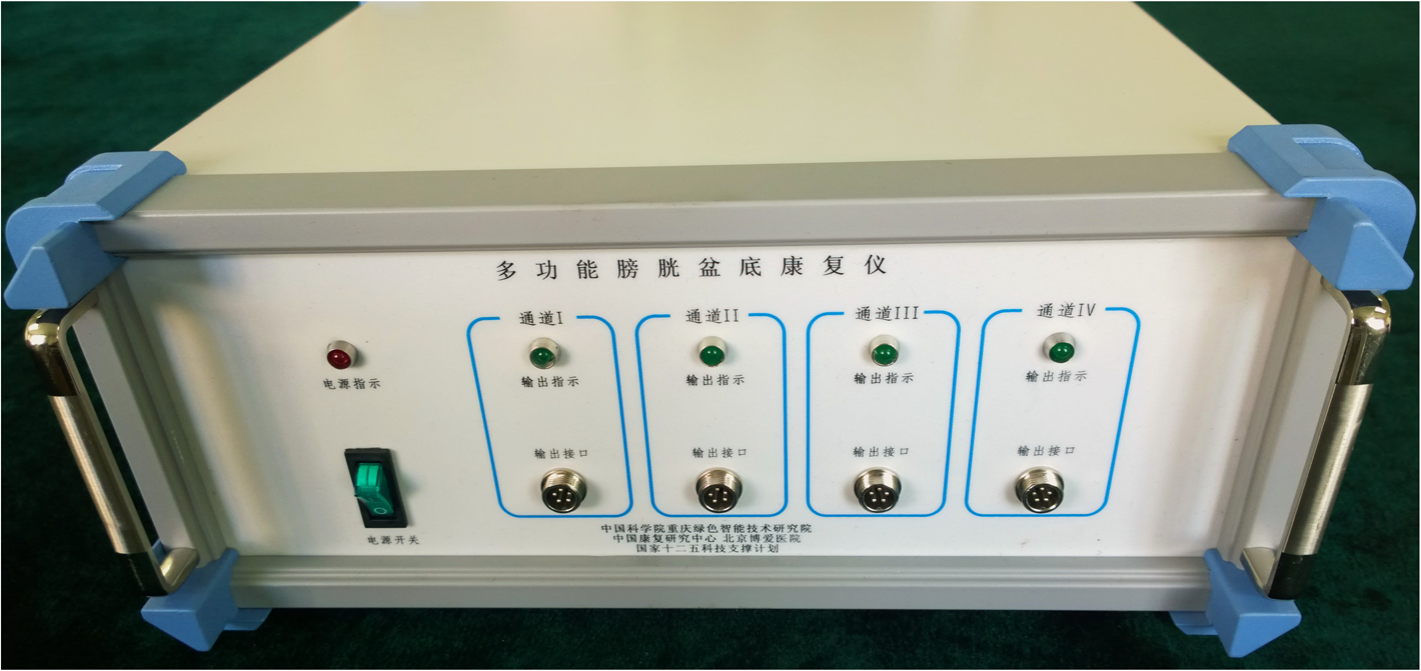
**The picture of the Bladder-Pelvic Stimulator (I).**

The Bladder-Pelvic Stimulator (I) consists of three sub-systems (stimulation circuit, user interface device, and electrode adaptor; Figure 1). The stimulator supports four independent output channels. The stimulator can generate mono- and bi-polar pulse wave with user-defined waveforms and parameters as follows (step sizes are given in parentheses): pulse amplitude 1–50 mA (0.5 mA); pulse frequency 1–100 Hz (1 Hz); pulse width 50–2000 μs (10 μs); pulse train duration 0.1 – 10 s (0.1 s); pulse train interval 0 – 10s (0.1 s); pulse train rising edge 0 – 10s (0.1 s); and pulse train falling edge 0.01 – 10 s (0.1 s). The user interface was developed on the android tablet platform. Clinicians can select and modify stimulation parameters on the tablet. The electrode interface supports multiple types of implantable and surface electrodes, which were not included in this study.

The stimulation applied in this study was a continuous, bipolar square wave form with a pulse duration of 200 μs and a stimulation frequency of 5 Hz. The stimulator was controlled to determine the minimal current needed to induce a toe twitch. The stimulation intensity was then increased to the maximal level, which was comfortable for the subject during the entire 30-min stimulation. The volume per CIC was averaged among subjects during 5 periods, as follows: 1) 48–24 hours before foot stimulation; 2) 24 hours before foot stimulation; 3) up to 24 hours after stimulation; 4) 24–48 hours after stimulation; and 5) 48–72 hours after stimulation.

One-way ANOVA, followed by the Dunnett multiple comparison test, was used to detect statistically significant differences (p <0.05) between voided volumes before and after stimulation.

## Results

The baseline characteristics and the stimulation intensities of the patients are shown in Table 1.

**Table 1:** **The baseline characteristics and the volume per CIC before and after stimulation**

**Subject-sex-age no.**	**Neurological pathology**	**Visiting time (months)**	**Stimulation intensity (mA)**		**Mean ± SE Vol/CIC (ml)**				
			**Left**	**Right**	**48 ~ 24 h before**	**In 24 h before**	**Up to 24 h after**	**24 ~ 48 h after**	**48 ~ 72 h after**
1-M-26	Incomplete spinal cord injury	1	15	20	263.3 ± 68.4	266.7 ± 66.7	300.0 ± 57.7	266.7 ± 44.1	270.0 ± 65.1
2-M-43	Incomplete spinal cord injury	3	50	50	310.0 ± 5.8	305.0 ± 9.6	410.0 ± 23.8	290.0 ± 5.8	302.5 ± 13.2
3-M-17	Spina bifida	1	30	30	190.0 ± 4.2	191.7 ± 4.4	246.0 ± 18.3	210.0 ± 7.7	200.0 ± 4.1
4-F-17	Meningocele	1	20	20	291.7 ± 41.7	286.7 ± 21.1	400.0 ± 60.2	358.0 ± 58.9	253.3 ± 36.9
5-M-38	Meningocele	3	15	15	246.7 ± 42.2	260.0 ± 19.2	302.9 ± 21.8	240.0 ± 24.7	258.3 ± 33.5
6-F-17	Meningocele	6	20	15	300.5 ± 33.7	315.0 ± 26.8	385.5 ± 41.2	320.0 ± 38.4	305.8 ± 29.1
7-M-46	Incomplete spinal cord injury	3	22	25	286.0 ± 62.3	300.0 ± 35.8	344.0 ± 50.4	325.0 ± 41.1	253.3 ± 27.7
8-F-24	Meningocele	3	30	27	266.0 ± 15.7	271.4 ± 19.6	364.0 ± 45.8	291.7 ± 14.2	261.7 ± 25.6
9-M-38	Incomplete spinal cord injury	12	50	50	310.0 ± 17.0	328.6 ± 19.6	424.0 ± 31.2	325.0 ± 41.1	320.0 ± 13.7
10-F-22	Spina bifida	6	18	20	276.0 ± 12.9	285.7 ± 12.3	352.0 ± 8.6	281.7 ± 5.4	265.0 ± 5.6
11-M-30	Meningocele	12	25	20	334.0 ± 6.8	328.6 ± 19.6	444.0 ± 11.7	341.7 ± 11.7	336.7 ± 14.1

**Figure 2:**
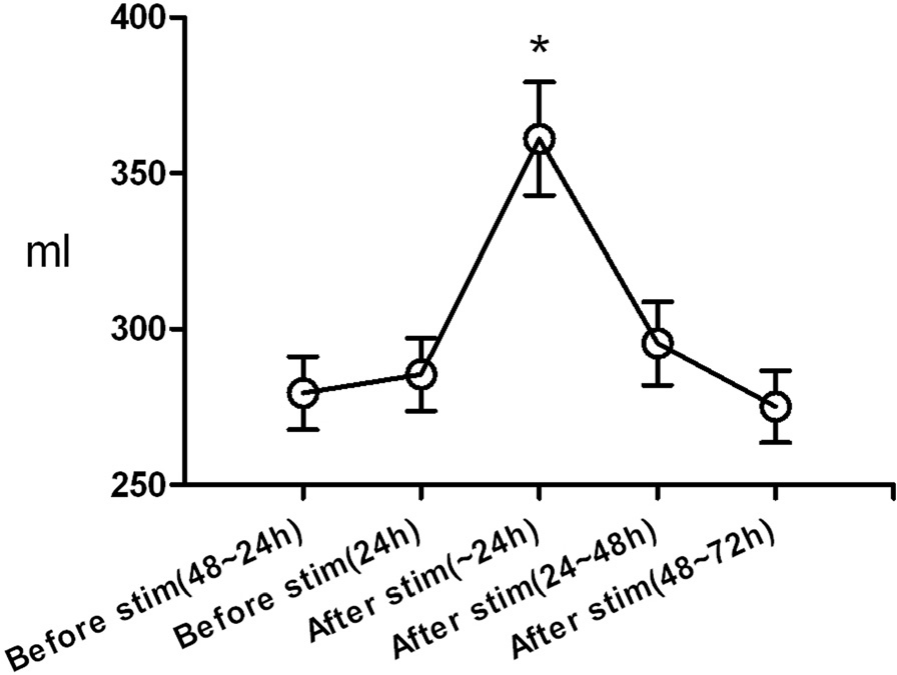
**Mean bladder volume per CIC measured in 11 subjects during 48–24 h, 24 h before foot stimulation (Stim), and within 24 h, during 24–48 h and 48–72 h after foot stimulation at 5 Hz frequency, 200 μs pulse width, and 15–50 mA intensity.** Asterisks indicate significantly different versus voided volume before stimulation (p <0.05).

The volume per CIC was 279.4 ± 11.7 ml and 285.4 ± 11.8 ml during the 1st and 2nd periods, respectively. During the 3rd period, the average volume per CIC increased to 361.1 ± 18.1 ml after stimulation (p <0.05; Figure 2). The average volume per CIC returned to 295.4 ± 13.4 ml and 275.1 ± 11.5 ml in the 4th and 5th periods, respectively.

The volume per CIC remained increased for 24 h after stimulation in all of the patients. Subjects 4 and 7 had greater bladder capacities 24–48 h after stimulation than before stimulation. Forty-eight hours after stimulation, the volume per CIC returned to pre-stimulation baseline in all patients. Subject 3, who was 1 month post-surgery, felt a desire to void when the capacity was approximately 190 ml, but the bladder filling sensation was delayed and the bladder volume was increased to 246.0 ± 18.3 ml after stimulation.

All subjects tolerated stimulation without discomfort. There were no immediate or long-term adverse events associated with stimulation.

## Discussion

In the current study all of the patients underwent sigmoid cystoplasties for 1–12 months (mean, 4.6 ± 1.2 months) because of a neurogenic bladder secondary to incomplete spinal cord injuries, meningoceles, or spina bifida. After recovering from surgery, the patients were asked to empty their bladders by CIC; however, the mean bladder capacity was only 280.5 ± 11.7 ml at early follow-up post-operatively based on urodynamic evaluation, which was unsatisfactory for those subjects who had undergone cystoplasty. In our previous research [1], we showed that the most common problem within 6 months post-operatively was incontinence, which might have resulted from the weakened function of the sphincter post-operatively. The presence of an indwelling urethral catheter for a long period could contribute to sphincter weakness because the maximal urethral pressure at rest was significantly decreased during the 6-month follow-up examination compared with the pre-operative pressure. Automatic contraction of the intestinal reservoir could also lead to pressure increase and incontinence. The residual detrusor also may maintain DO post-operatively. New bladder wall edema can result in reduced bladder compliance. The aforementioned four reasons can explain why the bladder capacity of the patients in the current study was not satisfactory at the early follow-up evaluation. The patients continued to use CIC combined with oral anti-cholinergic agents until the bladder capacity became larger 6 months post-operatively. The anti-cholinergic agents have some side effects, e.g., dry mouth, thus the patients cannot take the medications for a long time.

Previous studies in cats showed that transcutaneous electrical stimulation of somatic afferent nerves in the foot inhibits reflex micturition, significantly increases bladder capacity [5], and induces post-stimulation inhibition of reflex bladder activity that persists for 1–2 h [2]. Indeed, the same mechanisms might occur in healthy humans. It has been demonstrated that transcutaneous electrical stimulation of somatic afferent nerves in the foot can delay bladder filling sensations and significantly increase bladder capacity > 50% in healthy humans, and this technology has the potential to be an effective new treatment for patients with DO [3].

In our study, foot stimulation using skin surface electrodes also can delay the bladder filling sensation and significantly increase bladder capacity (Table 1 and Figure 1) in the patients after sigmoid cystoplasty. The volume per CIC was significantly increased compared with baseline, and this effect can last > 1 day.

The mechanism underlying foot stimulation is unknown, but may be mediated by the nerve in the foot [3]. The stimulation electrodes were placed on the skin surface rather than directly on the nerves. Which nerves were activated? The tibial nerve courses from the inner ankle inferiorly to the plantar surface of the foot and branches into the lateral and medial plantar nerves at the location of the electrodes. These nerves further branch into multiple small nerves that course toward the toes. Thus, it is highly likely that foot stimulation activates afferent branches of the tibial nerve in the lateral and medial plantar aspects of the foot.

The spinal segmental distribution of the stimulated somatic afferent pathways is an important factor in the efficacy of this type of neuromodulation [5]; however, inhibition at a supraspinal site cannot be excluded. A previous study in cats showed that the inhibitory effect on bladder activity elicited by electrical stimulation of the nerves from the hind limb muscles was lost after chronic spinal cord transection at the thoracic level, indicating a possible role of the supraspinal mechanisms in somato-vesical inhibition [6]. In the current study, all of the patients had incomplete spinal cord damage; therefore we cannot confirm whether or not foot stimulation has the same effects in patients with complete spinal cord damage. The Chen study [3] showed that some subjects voided a larger volume after only 30 minutes of stimulation, indicating that 30 minutes of stimulation might be sufficient to induce an inhibitory effect; thus, foot stimulation was applied for only 30 minutes in the current research.

In the Chen study [3], the average voided volume increased by > 50% or approximately 200 ml, which is more than the increase (approximately 30%) in volume per CIC in the current study. We calculated the mean volume per CIC in 24 hours after stimulation during the 3rd period, which was > 5 hours in the Chen study [3]. It is well known that the stimulation effect will weaken over time. Thus, if we also calculate the average volume per CIC 5 h after stimulation, the result may be close to the volume reported by Chen [3].

Although only a few subjects with neurogenic bladder secondary to incomplete spinal cord injuries, meningoceles, or spina bifida were tested in the study who used CIC to empty the bladder post-sigmoid cystoplasty, our results support proceeding with clinical trials involving foot stimulation in patients with OAB and other types of neurogenic bladder. Currently, CIC combined with an anti-cholinergic medication is the gold standard treatment for NDO; however, many patients are refractory to the medication or have dose-limiting side effects [7]. If foot stimulation can inhibit DO, improve bladder compliance, and increase bladder capacity in patients with neurogenic bladder, foot stimulation can be used to treat the patients instead of anti-cholinergic medications.

This is the first clinical trial in which electrical stimulation of the foot was used to treat patients. We want to determine whether or not this treatment can increase the bladder capacity in patients. Although a positive effect was shown in the current study, there were some flaws and limitations in the study. In the future, we need to conduct a randomized controlled trial to further elucidate and confirm our findings. First, the subjects in the current study all had meningoceles, incomplete spinal cord injuries, or spina bifida who had undergone sigmoid cystoplasties for 1–12 months and they do not represent all types of lower urinary tract disorders, thus we need to continue investigating patients with OAB and other types of neurogenic bladder. Second, we only focused on the changes of volume per CIC; CIC times and the urodynamic data after stimulation require study to verify the effect. Third, additional studies with a larger number of subjects are required to determine the optimal stimulation duration/pattern and further elucidate the post-stimulation effect. Fourth, the neobladder is composed of the residual bladder and sigmoid, still it is not known whether the capacity increased due to residual bladder or the sigmoid from the current data. It is difficult to prove in an augmentation model unless a pre-AE stim response is also recorded; alternatively, a similar study on orthotopic neobladder can also answer the question of whether detubularized bowel can respond to peripheral stimulation. However, no pre-augmentation stim was performed in this study. Though it is verified that neuromodulation is effective for both bladder and bowel dysfunction in previous literature [8], we still need to perform some studies to answer above-mentioned questions in the future. Fifth, all of the patients underwent this procedure at our medical center, but we are designing a portable bladder-pelvic stimulator so that patients can operate it in their own homes.

## Conclusions

Electrical stimulation of the foot using skin surface electrodes can delay the bladder filling sensation and significantly increase bladder capacity in neurogenic bladder patients after sigmoid cystoplasty. Thus, foot stimulation will be a promising treatment option if it is shown to be clinically effective in OAB and NDO in the future because it is non-invasive and can be easily managed by patients.

## Abbreviations

SNM: Sacral neuromodulation
PNS: Pudendal nerve stimulation
OAB: Overactive bladder
NDO: Neurogenic detrusor overactivity
TNS: Tibial nerve stimulation
DO: detrusor overactivity
CIC: Clean intermittent catheterization
ASIA: American Spinal Injury Association
